# Skp2 and Skp2B team up against Rb and p53

**DOI:** 10.1186/1747-1028-6-1

**Published:** 2011-01-21

**Authors:** Doris Germain

**Affiliations:** 1 Mount Sinai School of Medicine, Tisch Cancer Institute, Division of Hematology/Oncology, One Gustave L. Levy Place, Box 1079, New York, New York 10029, USA

## Abstract

The Skp2 locus encodes two proteins, Skp2 and Skp2B. The role of Skp2 in the ubiquitin-dependent degradation of key regulators of the retinoblastoma protein pathway has been well established. More recent work from the McCormick's group suggested that Skp2 has an ubiquitin-independent function in the regulation of the p53 pathway. Adding to this observation, we reported that Skp2B also regulates the activity of p53 by degrading a distinct substrate, prohibitin. Since prohibitin has been implicated in the regulation of the Rb pathway, collectively, these observations suggest that Skp2 and Skp2B team up against p53 and Rb.

## Introduction

The retinoblastoma (Rb) and the p53 pathways are two major mechanisms of tumor suppression. Disruption of these pathways is observed in most cancers. This review focuses on the description of two mechanisms by which both Rb and p53 pathways are disrupted simultaneously. First, the loss of the well known p16INK4a/p19ARF locus and second, the less well known amplification of the Skp2/Skp2B locus.

### The p16INK4a/p19ARF locus regulates the Rb and p53 pathways

The remarkable genetic organization of some loci suggests that evolution has selected mechanisms to maximize their biological impact. One example of such clever organization is the p16^Ink4a^/p19^Arf ^locus. By using two distinct promoters, this locus allows the expression of two different proteins using overlapping genetic material (reviewed in [1]; the p16 protein, a cyclin-dependent kinase (cdk) inhibitor and ARF, an indirect regulator of the tumor suppressor gene p53.

The cyclin dependent kinases (cdk) are a family of protein serine/threonine kinases, which control cell cycle progression through association with their regulatory subunits, known as cyclins. Cyclins are classified into a large number of subtypes including the D, E, A and B-type cyclins. Humans encode three D-type cyclins, cyclin D1, cyclin D2 and cyclin D3. D-type cyclins associate with cdk4 and 6 to promote the phosphorylation of the Retinoblastoma (Rb) protein (for review, [2]). Rb forms a complex with the E2F family of transcription factors and this represses their activity. Hyperphosphorylation of Rb results in the release of E2F, which then activates transcription of genes required for DNA replication and entry into S phase [3]. One of the early targets of E2F mediated transcription is cyclin E that, together with cdk2, acts to maintain Rb phosphorylation.

Cyclin-cdk complexes are themselves regulated by two families of cdk inhibitors including p27 of the p21 family, which inhibits cyclin E-cdk2 complexes [4], and p16 of the INK4 family, which inhibits cyclin D-cdk4/6 complexes [5]. Functional disruption of the tumour suppressors p16^INK4a ^or Rb or overexpression of cyclin D1 and CDK4 is frequently observed in many cancer types suggesting that disrupting the 'Rb pathway' is an essential part in oncogenesis [6].

The disruption of the p53 tumor suppressor is also an essential part of oncogenesis and p53 mutation is one of the most frequent genetic aberration observed in cancer. However, in addition to mutations, other mechanisms have evolved to disrupt this pathway. For example, disruption of ARF, allows for the accumulation of the ubiquitin ligase mdm2, which results in the degradation and inactivation of p53. Therefore, the loss of the p16INK4a/p19ARF locus allows for the simultaneous disruption of both the Rb and p53 pathways.

### Overexpression of Skp2 regulates the Rb and p53 pathways

More recently the overexpression of the F-box protein Skp2 was found to mediate an alternative mechanism leading to the disruption of the Rb and p53 pathways. F-box proteins act as the substrate recognition subunits of specific ubiquitin ligase complexes. Linkage of ubiquitin to a protein is a highly organized process involving the sequential action of an ubiquitin-activating enzyme (E1), an ubiquitin-conjugating enzyme (E2) and an ubiquitin-ligase (E3). When this enzymatic cascade results in the attachment of a lysine 48 polyubiquitin chain onto a substrate, it serves as a signal for degradation by the 26S proteasome. Most of the regulation of the ubiquitination pathway occurs at the level of the ubiquitin ligase. Of particular interest to this review is the ubiquitin ligase complex termed the SCF complex [7-10] that is composed of Skp1, a cullin, an F-box protein and the ring finger protein Roc-1[11-13]. F-box proteins act as adaptors by associating with substrate proteins, bringing them to the core of the SCF by binding to Skp1 [14]. The SCF^Skp2 ^complex refers to the SCF in which Skp2 is the F-box protein. The overexpression of Skp2 has been linked to the progression of several tumors due to its involvement in the degradation of a key regulator of the cell cycle.

Skp2 is involved in the ubiquitin-dependent degradation of the Cyclin-dependent kinase (Cdk) inhibitor p27 [15,16]. p27 binds to, and inhibits Cyclin E-Cdk2 complexes, and as a result, prevents progression through the cell cycle. Upon phosphorylation of threonine-187, p27 binds to Cks1 and Skp2 [17], it becomes ubiquitinated and subsequently degraded. Consequently, the degradation of p27 leads to the activation of Cyclin E-Cdk2 complexes and hence entry into the S-phase of the cell cycle. Loss of p27 is a frequent event in several cancer types and is associated with poor prognosis [18-20]. However, the lack of p27 expression is not due to the loss of the p27 gene, but rather due to the overexpression of Skp2 [19].

Skp2 was more recently shown to attenuate the p53 pathway [21] and this function appears to be independent of SCF-mediated proteolysis. This was revealed when an F box-deleted version of Skp2, which cannot bind to the SCF, was found to be equally potent as an attenuator of p53. Kitagawa et al, described that Skp2 binds the transcriptional co-activator p300, therefore blocking the interaction between p300 and p53. As acetylation of p53 by p300 is essential for its activation [22] Skp2 inhibits the transactivation function of p53.

Therefore, as observed with the loss of the p16Ink4a/p19Arf locus, overexpression of Skp2 leads to the disruption of both the Rb and p53 pathways.

### Skp2 is not alone, the contribution of Skp2B to the deregulation of Rb and p53 pathways

Our study of Skp2B recently contributed a new twist to this story. Like other F-box proteins such as b-TRCP, Skp2 has been reported to have three alternative splice forms, Skp2 or Skp2A, Skp2B and Skp2-gamma, although Skp2-gamma remains uncharacterized.

**Figure 1:**
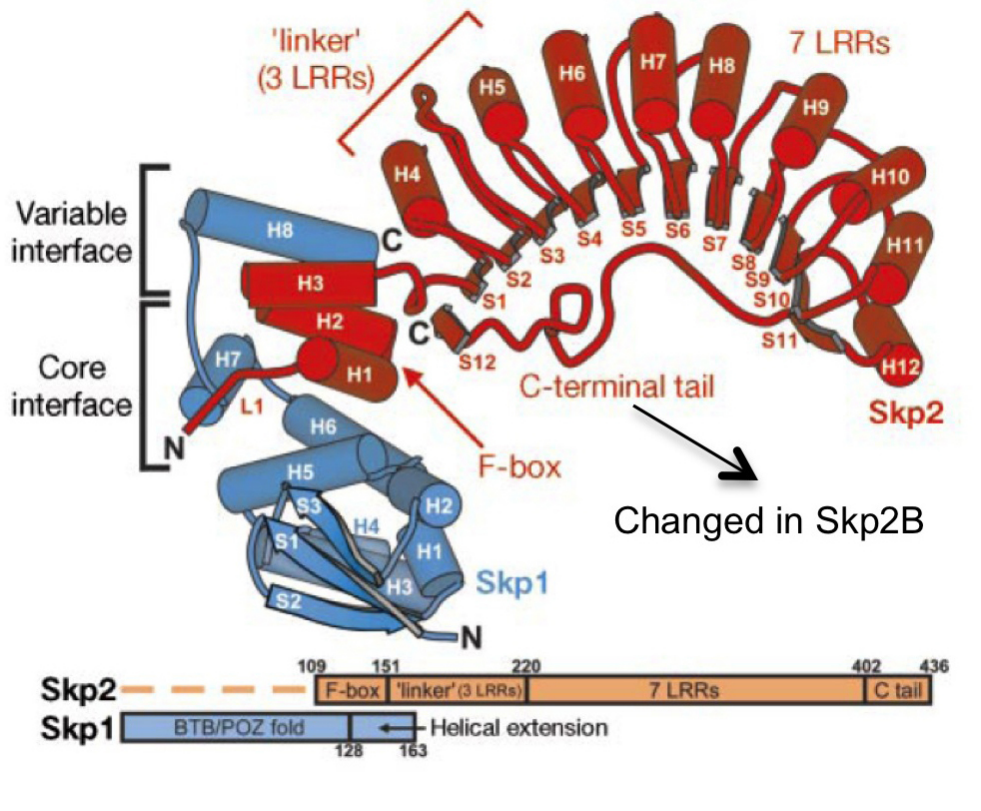
**Structure of Skp2 as determined by Schuman et al, 2000**. The C-terminal domain is encoded by a different exon in Skp2B.

Skp2 has three important domains: an N-terminal F-box motif, required for binding to Skp1, followed by 10 consecutive leucine rich repeats (LRR), located near the C-terminus and required for binding to substrates (Figure 1). The third important domain is the C-terminus, as it folds back near the F-box and stabilizes the interaction with Skp1 (Figure 1).

We identified Skp2B in 2001, and called it originally Skp2-CTV (C-Terminal Variant) as it is characterized by the presence of a unique C-terminal domain (Figure 1) [23]. We previously reported that Skp2A and Skp2B are both overexpressed in breast cancers [24]. We found that Skp2B differs from Skp2 in several respects, notably that Skp2B is cytoplasmic while Skp2 is nuclear, and that, unlike Skp2, Skp2B does not regulate p27 levels [24].

Interestingly, since the isolation of Skp2B, a number of studies using antibodies that recognize both Skp2 and Skp2B have described the presence of strong cytoplasmic staining in various cancer types, including breast cancer [18], although the percentage of cytoplasmic versus nuclear staining was not reported in this breast cancer study. In prostate cancer within the African-American population, however, cytoplasmic staining was found to be predominant. Moreover, the lack of correlation between Skp2 and p27 levels in a large number of these cases led the authors to conclude that Skp2 expression in prostate cancer pathogenesis might not be exclusively related to p27 degradation [25]. One obvious explanation is that the cytoplasmic form is Skp2B, for which p27 is not a substrate. In support of this conclusion, two additional studies, one in Kaposi's sarcoma, and another in cervical cancer, found no correlation between Skp2 expression and loss of p27. Further, both of these studies reported a significant percentage of cytoplasmic staining, which also suggests that Skp2B overexpression in these tumors may explain their observations [26,27].

In order to determine the role of Skp2B in breast cancer, we established transgenic mice expressing Skp2B under the mammary-tumor virus (MMTV) promoter. These mice displayed an accelerated invasion of the mammary fat pad by the mammary tree during puberty, an accelerated lobulo-alveolar development, which is normally restricted to pregnancy, and gross cyst formation. In addition, they also developed mammary tumors of varying histology, including high-grade adenocarcinomas with undifferentiated pattern [28]. The formation of tumors was greatly accelerated by pregnancy suggesting a synergy between the pathways activated by Skp2B overexpression and those activated during pregnancy.

In parallel to establishing transgenic mice, we performed a 2-hybrid screen using the C-terminal domain that is unique to Skp2B as the bait and identified two proteins containing an SPFH domain (Stomatin/Prohibitin/Flotillin/HflK/C). SPFH proteins have been implicated in a variety of functions including as chaperones, and in senescence and proliferation. The two SPFH proteins isolated as Skp2B associated proteins are the repressor of the estrogen receptor activity (REA), also known as prohibitin-related and prohibitin.

Among the co-repressors, the repressor of estrogen receptor activity (REA) has profound effect on the development of the mammary gland [29,30]. REA heterozygote mice display accelerated invasion of the fat pad and development of the mammary gland during pregnancy, which is associated with an increase in the ER activity [30].

We reported that Skp2B co-immuoprecipitates with REA and that Skp2B overexpression results in a decrease in REA levels and an elevation in the activity of the estrogen receptor [28]. Our data suggest that since MMTV-Skp2B transgenic mice display a phenotype closely related to that observed in REA heterozygote mice [30], Skp2B represents a novel regulator of the ER and, that Skp2B overexpression in primary breast cancer, plays a significant role in breast cancer. However, since REA heterozygote mice do not develop mammary tumors while MMTV-Skp2B mice do, this observation suggested that Skp2B must have additional substrates.

We later reported that the over-expression of Skp2B promotes the ubiquitination and subsequent degradation of prohibitin [31]. Since Prohibitin binds and stimulates the transcriptional activity of p53 [32], we reasoned that lost of prohibitin, as a result of Skp2B overexpression, may disrupt p53 activity and contribute to the formation of mammary tumor in the MMTV-Skp2B transgenic mice [31]. In support of this hypothesis we reported that the activity of p53 is attenuated by the overexpression of Skp2B both *in vitro *and *in vivo *[31].

Of relevance to this review is the additional finding that prohibitin represses the transcriptional activity of E2F [33-37]. Mechanistically, prohibitin binds to Rb, Brg-1 and Brm, which are recruited to E2F responsive promoters by prohibitin to repress E2F-mediated transcription. While the recruitment of Brg-1 and Brm by prohibitin is independent of Rb, the prohibitin-Brg-1-Brm repression of E2F required Rb, suggesting that prohibitin acts to enhance the repressive effect of Rb.

However, it is important to note that a wide number of distinct functions have been attributed to prohibitin and a fundamental divergence of opinion regarding the role of prohibitin in cancer exists. On one hand, strong evidence suggests that prohibitin function is essential to maintain mitochondrial function and cellular proliferation [38-42] and that prohibitin is overexpressed in cancer [39]. On the other hand, a large number of studies indicate that prohibitin acts as a tumor suppressor and prevents cellular proliferation [32,34,36,37,43-45]. While this discrepancy appears difficult to reconcile, one key distinction between these studies is that prohibitin is reported to be present in multiple cellular locations. In the mitochondria, prohibitin is found to be required for proliferation, while prohibitin in the nucleus appears to inhibit a number of transcription factors such as the estrogen receptor and the androgen receptor, or to stimulate the growth inhibitory transcription factor p53. Therefore, if correct, these observations would suggest that in cancer, maintenance of prohibitin in the mitochondria and loss of its nuclear function are both required for growth.

We therefore propose that the differential regulation of nuclear and mitochondrial fraction of the prohibitins by the proteasome may offer a potential mechanism to reconcile the apparent discrepancies concerning the role of prohibitins in cancer.

### Skp2 and Skp2B: partners in crime

**Figure 2:**
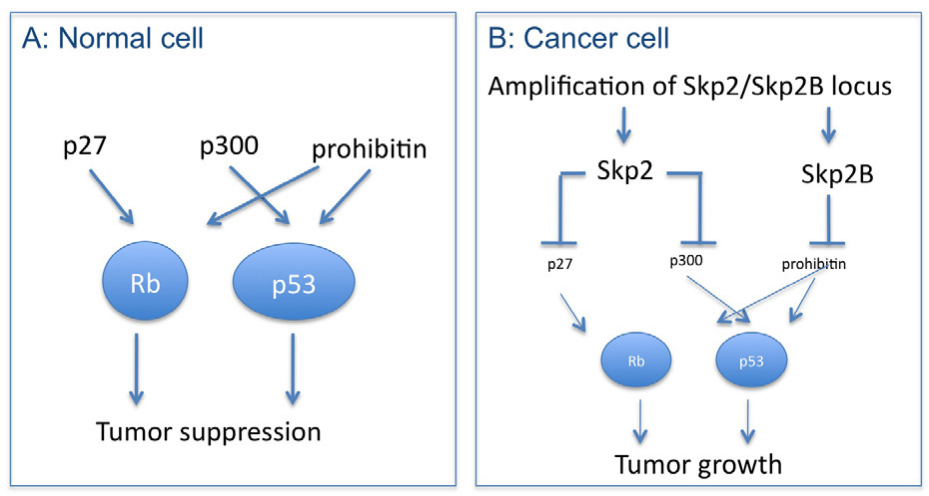
**Skp2 and Skp2B in normal and cancer cells.** (A) In normal cells, the Rb and p53 pathway prevents abnormal cell growth. The actions of p27, p300 and prohibitin assist Rb and p53 in their tumor suppressor functions. (B) In cancer cells, where the Skp2 and Skp2B locus are amplified, the functions of p27, p300 and prohibitin are lost and as a result, the Rb and p53 pathways are disrupted allowing cell growth.

Since Skp2 and Skp2B overexpression in primary breast cancers is not mutually exclusive [24], we propose a model for the combined effect of Skp2 and Skp2B (Figure 2). In this model by degrading their respective substrates Skp2 and Skp2B affect simultaneously the Rb and the p53 pathways and therefore the amplification of the Skp2/Skp2B loci represents a powerful mechanism of oncogenesis. These observations raise the possibility that even in the absence of p53 mutations or mdm2 amplification, the activity of p53 may be attenuated due to the amplification of the Skp2 locus. Further, since Skp2 overexpression was reported to cause resistance to the anti-estrogen drug tamoxifen [18,46] and estrogen stimulates Skp2 expression [46], while we found that Skp2B promotes the activity of the estrogen receptor [28], the combined effect of both Skp2 and Skp2B is predicted to also activate this pathway. These combined actions may underline the worst prognosis associated with Skp2/2B over-expression [21]. Therefore, as for the lost of the p16INK4a/p19ARF locus, amplification of the Skp2/Skp2B locus represents an alternative mechanism of disruption of the Rb and p53 pathways. Since Skp2 and Skp2B differ only at the C-terminal domain but are otherwise identical, molecules able to inhibit both isoforms are predicted to be the most promising.
